# Sport Involvement Among Middle-Aged and Older Adults With Arthritis: Motivations and Constraints to Participation

**DOI:** 10.1093/geroni/igaa057.1303

**Published:** 2020-12-16

**Authors:** Megan Janke, Julie Son, Jill Naar, Stephanie West, Toni Liechty, Jen Wong

**Affiliations:** 1 East Carolina University, Mount Berry, Georgia, United States; 2 University of Idaho, Moscow, Idaho, United States; 3 Appalachian State University, Boone, North Carolina, United States; 4 University of Illinois, Champaign, Illinois, United States; 5 The Ohio State University, Columbus, Ohio, United States

## Abstract

Although participation in physical activity is recommended for adults with arthritis, research indicates individuals often stop participating in sports and physically active leisure due to the pain and symptoms associated with arthritis (Wilcox et al., 2006). Examining a group of older adults with arthritis, the present study examines motivations and constraints related to participating in sport and physically active leisure as well as how they negotiate constraints. Data (N=1203) were collected through an online questionnaire of adults aged 50 and older in the United States. This study includes individuals reporting a diagnosis of some form of arthritis (n=288; M age = 64.8, SD = 8.08). Approximately 32% self-reported participation in sport in the past 12 months. Descriptive statistics were conducted to explore motivations and constraints to sport involvement. Regressions were run to determine whether constraints and motivations explained adults’ functional mobility and social wellbeing. The most commonly identified motivation for participation was for health purposes (80.2%). Constraints to participation included not being in good enough shape (51.9%) and not having others their age with whom to participate (47.4%). The most commonly identified constraint negotiation was to budget money (51.4%); this is not surprising since sport participation was perceived as expensive (41.3%). Motivations (p<.01) and constraints (p<.001) significantly predicted functional mobility; constraints significantly predicted some aspects of social wellbeing (i.e., coherence, contribution, actualization; p<.05) while constraint negotiation predicted social acceptance (p<.05) and integration (p<.001). Discussion will include implications and strategies for agencies and professionals who work with adults who have arthritis.

